# Infertility in Fabry’s Disease: role of hypoxia and inflammation in determining testicular damage

**DOI:** 10.3389/fendo.2024.1340188

**Published:** 2024-02-22

**Authors:** Luigi Sansone, Federica Barreca, Manuel Belli, Michele Aventaggiato, Andrea Russo, Giulietta A. Perrone, Matteo A. Russo, Marco Tafani, Andrea Frustaci

**Affiliations:** ^1^ Laboratory of Cellular and Molecular Pathology, IRCCS San Raffaele, Rome, Italy; ^2^ Department of Human Sciences and Promotion of the Quality of Life, San Raffaele Roma Open University, Rome, Italy; ^3^ Laboratory of Cellular and Molecular Pathology, Department of Experimental Medicine, Sapienza University, Rome, Italy; ^4^ UOC of Pathologic Anatomy, IRCCS Regina Elena National Cancer Institute, Rome, Italy; ^5^ UOC of Pathologic Anatomy, San Filippo Neri Hospital, Rome, Italy

**Keywords:** Fabry’s disease, hypoxia, inflammation, cellular and molecular rehabilitation, testicular damage, new therapeutic targets for infertility rehabilitation

## Abstract

**Introduction:**

Fabry’s disease (FD) is a genetic X-linked systemic and progressive rare disease characterized by the accumulation of globotriaosylceramide (GB3) into the lysosomes of many tissues. FD is due to loss-of-function mutations of α-galactosidase, a key-enzyme for lysosomal catabolism of glycosphingolipids, which accumulate as glycolipid bodies (GB). In homozygous males the progressive deposition of GB3 into the cells leads to clinical symptoms in CNS, skin, kidney, etc. In testis GB accumulation causes infertility and alterations of spermatogenesis. However, the precise damaging mechanism is still unknown. Our hypothesis is that GB accumulation reduces blood vessel lumen and increases the distance of vessels from both stromal cells and seminiferous parenchyma; this, in turn, impairs oxygen and nutrients diffusion leading to subcellular degradation of seminiferous epithelium and sterility.

**Methods:**

To test this hypothesis, we have studied a 42-year-old patient presenting a severe FD and infertility, with reduced number of spermatozoa, but preserved sexual activity. Testicular biopsies were analyzed by optical (OM) and transmission electron microscopy (TEM). Activation and cellular localization of HIF-1α and NFκB was analyzed by immunofluorescence (IF) and RT-PCR on homogeneous tissue fractions after laser capture microdissection (LCMD).

**Results:**

OM and TEM showed that GB were abundant in vessel wall cells and in interstitial cells. By contrast, GB were absent in seminiferous epithelium, Sertoli’s and Leydig’s cells. However, seminiferous tubular epithelium and Sertoli’s cells showed reduced diameter, thickening of basement membrane and tunica propria, and swollen or degenerated spermatogonia. IF showed an accumulation of HIF-1α in stromal cells but not in seminiferous tubules. On the contrary, NFκB fluorescence was evident in tubules, but very low in interstitial cells. Finally, RT-PCR analysis on LCMD fractions showed the expression of pro-inflammatory genes connected to the HIF-1α/NFκB inflammatory-like pathway.

**Conclusion:**

Our study demonstrates that infertility in FD may be caused by reduced oxygen and nutrients due to GB accumulation in blood vessels cells. Reduced oxygen and nutrients alter HIF-1α/NFκB expression and localization while activating HIF-1α/NFκB driven-inflammation-like response damaging seminiferous tubular epithelium and Sertoli’s cells.

## 1 Introduction

Fabry’s disease (FD) is a genetic X-linked systemic and progressive rare disease characterized by the deficiency or loss-of-function of α-galactosidase with accumulation of globotriaosylceramide (GB3) into the lysosomes of many tissues (1). GB3 accumulation is responsible for the damaging effects observed in heart, CNS, skin, kidney, endothelium, etc. (2–5). At a cellular level, GB3 accumulation has been linked to inflammation, ROS production, NFκB activation, leading to cell death as well as sclerosis, fibrosis, etc. (6–9). GB3 accumulation has also been observed in the testis (10). Here the results are scarce and sometime contradictory. In fact, both azoospermia, altered gonadal function as well as normal reproductive fitness have been reported (11–13). Moreover, FD patient presents normal hormonal levels (14). GB3 accumulation and lamellar or myelin-like bodies were described in Leydig’s cells, muscle cells, blood vessels, etc. Such observation leads to the conclusion that impairment of testicular function in FD patients is due to the progressive accumulation of GB3 in different cellular structures (5). However, so far there are no explanations for the pathogenetic pathway that, from GB3 accumulation leads to azoospermia and infertility. Our hypothesis is that GB3 accumulation in muscle cells, blood vessels, interstitial fibroblasts and stromal space alters oxygen and nutrients distribution and creates a pro-inflammatory response that alters seminiferous tubules microenvironment reducing spermatogenesis. To demonstrate our hypothesis and to provide a pathogenetic explanation, we performed ultrastructural (TEM) and molecular analysis (RT-PCR after LCMD) on testicular biopsies of a FD patient. Our results show that, compared to normal control testis, testicular tissue from FD patient has an accumulation of GB3 in vessel wall and in other stromal cells that may alter oxygen diffusion to both stroma and parenchymal tubular cells. Hypoxia triggers a molecular response of adaptation through the HIF-1α and NFκB activation, inducing hypoxic cell damage, an inflammatory state which can be cause of infertility.

## 2 Methods

### 2.1 Patients’ enrollment and characterization

In the present report, we have studied a 42-year-old patient presenting a systemic FD with cardiac (LV Maximal Wall Thickening 24 mm), renal (creatininemia 3.4 mg/dl), skin and eye (Cornea verticillata) involvement and large accumulation of GB3 (**Supplementary Figure S1**). Enzyme activity of α-galactosidase A (GLA) was 0.0% mM/mg/h (nv 20-65), due to gene mutation p.R227X causing a codon stop in Exon 5 of the GLA gene. Infertility with reduced number of spermatozoa (3.000.000/ml of poorly mobile sperm of semen) and preserved androgen levels (blood testosterone 680 ng/dL - NV 350-890 ng/dL; FSH 11 mUI/ml NV< 12 mUI/ml and LH 9.5 mUI/ml NV< 9 mUI/ml) and sexual activity.

As control, three fertile subjects, age 30-36 years (average: 33 years), undergoing testis micro-surgery, were enrolled. They were selected based on the absence of clinical and histopathological evidence of any proliferative and inflammatory process, and the presence of fertility, androgen levels and normal sexual activity. All patients had no prior long-standing anti-inflammatory treatment. Ethical approval was obtained from the Institutional Research Ethics Committee (Opinion number # 6/2019) (Institutional Review Board Statement: opinion number 6/2019) and all patients provided informed consent for the study.

### 2.2 Testicular biopsy

Testis specimens were collected immediately after biopsy or surgery. For molecular biology and immunofluorescence, each testis biopsy was snap-frozen in OCT compound with isopentane cooled in liquid nitrogen and stored at -80°C. Additional testis samples were fixed in glutaraldehyde for transmission electron microscopy (TEM) and for light microscopy (OM).

### 2.3 Histology and electron microscopy

For histological analysis, 3μm thick sections were stained with basic fuchsine to evaluate the presence of intracellular glycolipid as dark blue material in contrast with reddish cytoplasm and nucleus. As indicated by the present guidelines, “*disorders of the lysosomal type require electron microscopy for morphological diagnosis on tissue biopsy*” (15). For this reason, samples were fixed in 2% glutaraldehyde in 0.1 M phosphate buffer, at pH 7.3, post-fixed in osmium tetroxide 1,33% in the same buffer and processed following a standard schedule for embedding in Epon resin. Ultrathin sections were obtained by a diamond knife and stained with uranyl acetate and lead hydroxide. A Philips CM-10 TEM or Jeol 1400-plus TEM were used for observation and photographic analysis.

### 2.4 Laser capture microdissection (LSMD)

For each testicular biopsy, 8µm thick cryo-sections were prepared, tubular and interstitial tissue fractions were microdissected separately using a Nikon LCMD apparatus (Nikon, Amstelveen, The Netherlands). Microdissected tissue fractions were collected on the adhesive caps of the nanotubes and used for nucleic acid extraction (**Supplementary Figure S2**). Digital OM pictures were collected for each micro-dissected area if needed (**Supplementary Figure S2**).

### 2.5 RNA extraction and RT-PCR

LCMD samples were lysed to extract total RNA with the Ambion Paris system (Life technologies). After isolation, approximately 1 μg of RNA was reverse transcribed using the High-Capacity cDNA Archive Kit (Applied Biosystems, Milan, Italy) following manufacturer’s instructions. Aliquots of cDNA were subjected to real time PCR in 50 μl of 1 x Universal PCR Master Mix, 0.5 μM TaqMan probe and 5 ng of cDNA. Primers and for the genes studied were as follows: HIF-1α (forward: 5′-GATAGCAAGACTTTCCTCAGTCG-3′, reverse: 5′-TGGCTCATATCCCATCAATTC-3′); NFκB (forward: 5′-CCCACGAGCTTGTAGGAAGG-3′, reverse: 5′-GGATTCCCAGGTTCTGGAAAC-3′); VEGFA (forward: 5′-CCTCCGAAACCATGAACTTT-3′, reverse: 5′- ATGATTCTGCCCTCCTCCTT-3′); SAA1 (forward: 5′-TCGTTCCTTGGCGAGGCTTTTG-3′, reverse: 5′-AGGTCCCCTTTTGGCAGCATCA-3′); SAA2 (forward: 5′-CTGCAGAAGTGATCAGCA-3′, reverse: 5′-ATTATATGCATTATCTCAGC-3′); AGER (forward: 5′-CACCTTCTCCTGTAGCTTCAGC-3′, reverse: 5′-AGGAGCTACTGCTCCACCTTCT-3′); ALOX5 (forward: 5′-TGTGTGACTTGGGAGAGCTG-3′, reverse: 5′-TGTGTGCTAGGGGCTTTACC-3′); F2R (forward: 5′-CAAATGCCACCTTAGATCCCC-3′, reverse: 5′-CTTCTGAGA TGAATGCAGGAAGT-3′); IKBKB (forward: 5′-ACAGCGAGCAAACCGAGTTTGG-3′, reverse: 5′-CCTCTGTAAGTCCACAATGTCGG-3′); 18S (forward: 5′-GCAATTATTCCCCATGAACG-3′, reverse: 5′-GGGACTTAATCAACGCAAGC-3′). Each sample was loaded in duplicate, and negative and positive controls were included. Amplification of 18S rRNA was used as internal reference gene. PCR amplifications were performed using an ABI PRISM 7900 sequence detector (Applied Biosystems). Amplification data were analyzed using the Sequence Detector version 1.7 software (Applied Biosystems). Statistical analysis of real-time PCR results was performed using mean normalized cycle threshold (ΔCt) values and the pooled standard deviation of the mean ΔCt.

### 2.6 Immunofluorescence

Frozen tissue sections were fixed for 15 min with 4% paraformaldehyde (Immunofix, Bio-Optica, Milan, Italy; 05-K01015) in PBS, washed twice in PBS and permeabilized for 10 min with 0.5% Triton X-100 (Merck; X100) in PBS. After 1 h block with 1% bovine serum albumin (BSA, Merck; A3294) at room temperature, coverslips were incubated in a humidified chamber for 2 h at room temperature with a mouse anti-human HIF-1α (H1 alpha 67), (Novus Biologicals, LLC, Littleton CO, USA), rabbit anti-human NFκB p65, (Santa Cruz Biotechnology, Santa Cruz, CA), rabbit anti-human VEGFA ab46154, (Abcam, Cambridge, UK). Afterward, coverslips were washed with PBS 3 times (5 min/wash) and incubated for 1 h with goat anti-rabbit or goat anti-mouse IgG Alexa Fluor 555 fluorescent secondary antibody (1:200, Invitrogen, Carlsbad, CA, USA). Nuclei were stained with 100nM SYTOX™ green (Life Technologies, Thermo Fisher Scientific, Carlsbad, CA, USA). Finally, samples were washed with PBS 3 times (5 min/wash) and coverslips were mounted in ProLong Diamond Antifade Mountant (Life Technologies, Thermo Fisher Scientific, Carlsbad, CA, USA) and analyzed with an LSM510 confocal microscopy (Zeiss, Oberkochen, Germany). Quantification of the fluorescence intensity in the tubules and stroma of the control and FD patient was determined using Image J Software v1.51 (NIH, Bethesda, MD, USA).

### 2.7 Statistical analysis

Data are presented as the means and standard deviations determined from three or more experiments for condition. Differences between pairs of groups were analyzed by Student’s t-test. The level of significance was set at p < 0.05.

## 3 Results

### 3.1 Selective cellular GB3 accumulation in testicular biopsy from FD patient

**Figure 1:**
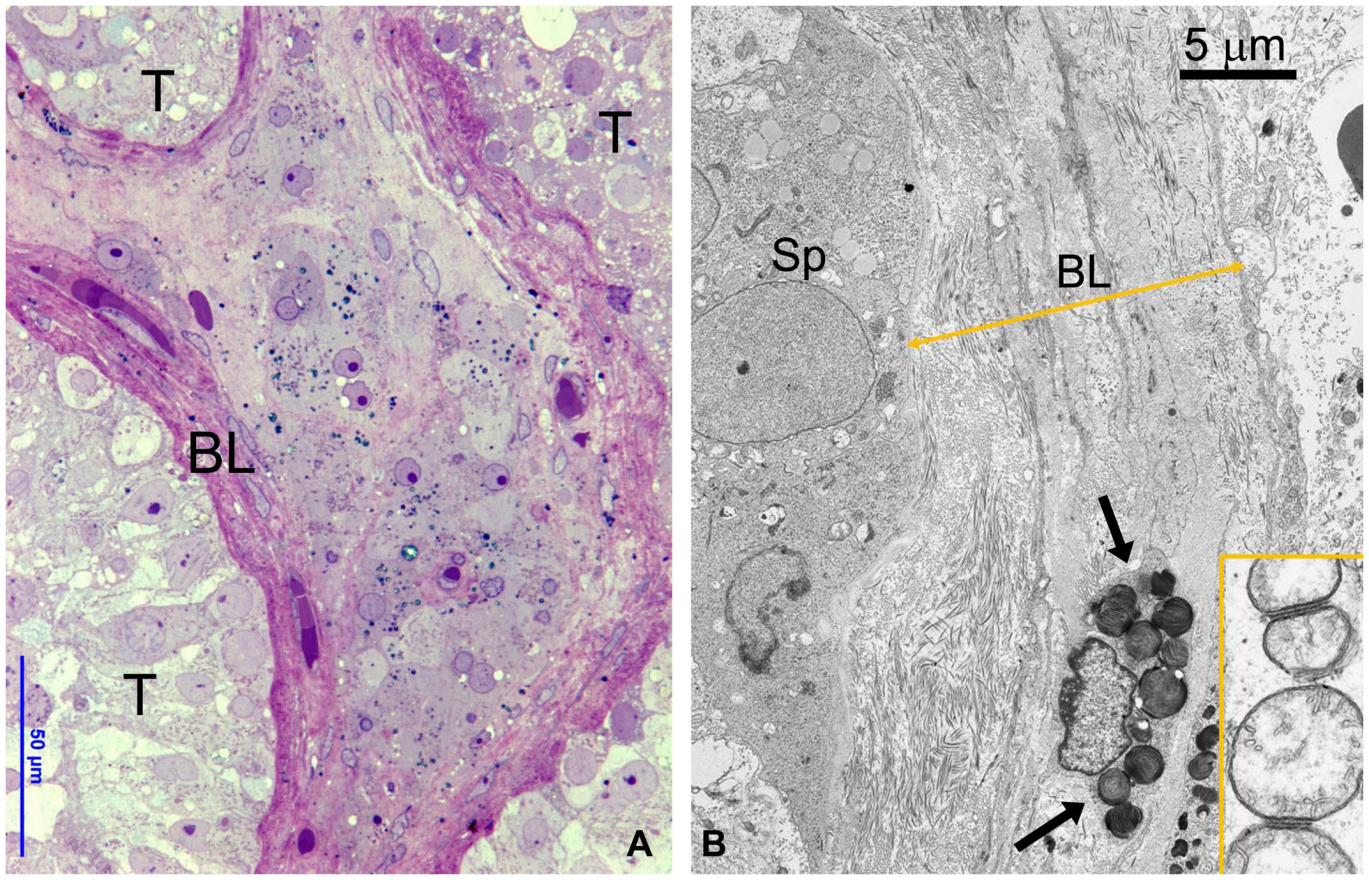
**(A)** OM showing part of three seminiferous tubules (T) without GB bodies and a central region of interstitial tissue with numerous GB bodies (dark blue deposits). To be noted the thickened layer of squamous smooth muscle cells around tubules. BL, basal layer. **(B)** TEM detailing the interface between the basal epithelium of seminiferous tubule and thickened basal layer (BL) around tubules layer (double arrowed yellow line). GB are present in the smooth muscle cells (arrows) and fibroblasts. Insert represents a detail of spermatogonial mitochondria interacting at level of outer membrane.

**Figure 2:**
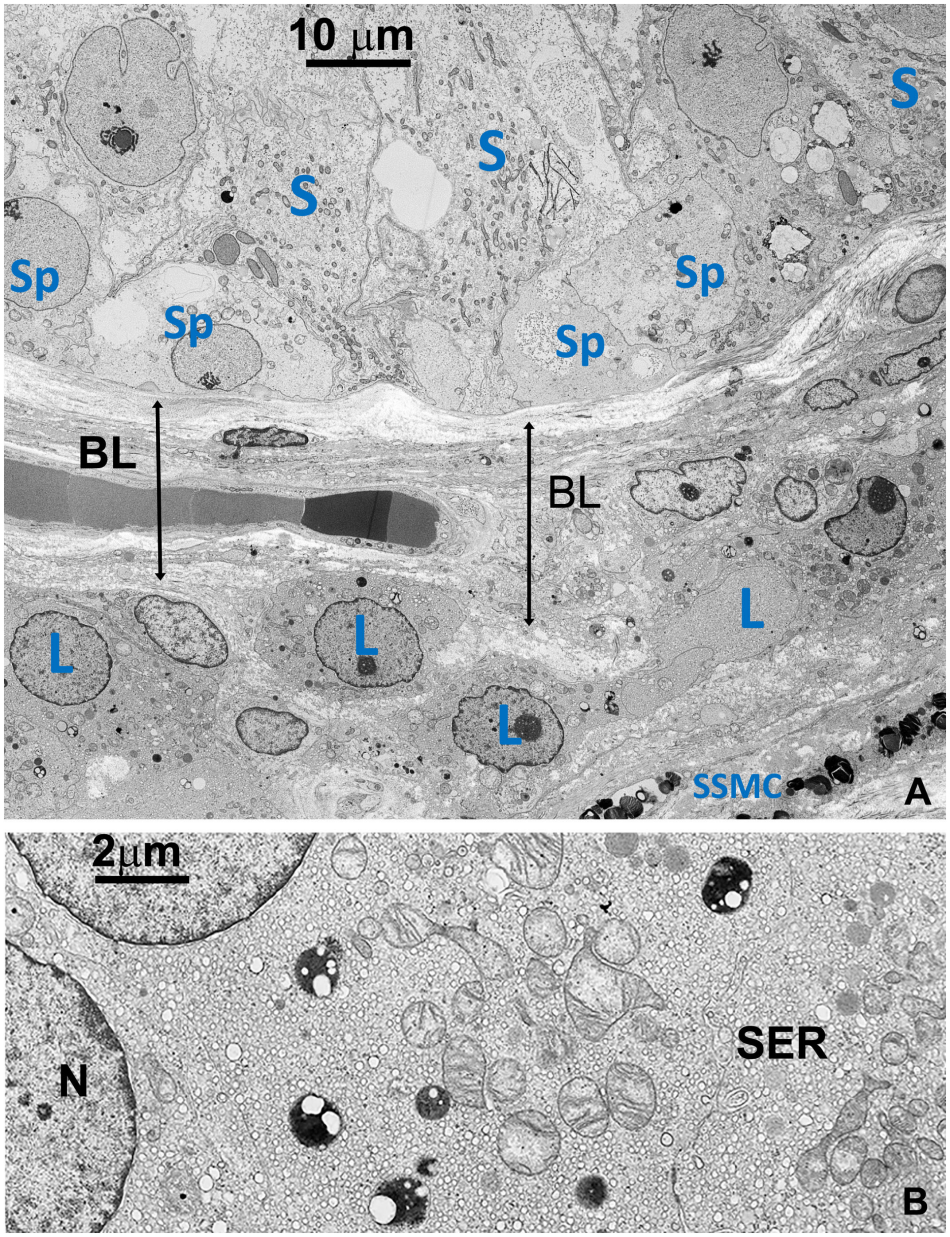
**(A)** TEM detailing the interface between the basal part of seminiferous tubule and the basal layer around tubules, including a small region of the stroma (right lower side). Sp, Spermatogonia; S, Sertoli’s cell; SSMC, Squamous Smooth Muscle Cell; L, Leydig cell; BL, basal layer **(B)** Detail of the cytoplasm of a Leydig cell (L). Most of the space is occupied by vesicular smooth endoplasmic reticulum (SER) typical of normal cells producing steroid sexual hormones. GBs are absent. A few small dense bodies are present in the cytoplasm which appears very different from GBs in their dimension and structure. N, nucleus.

**Figure 3:**
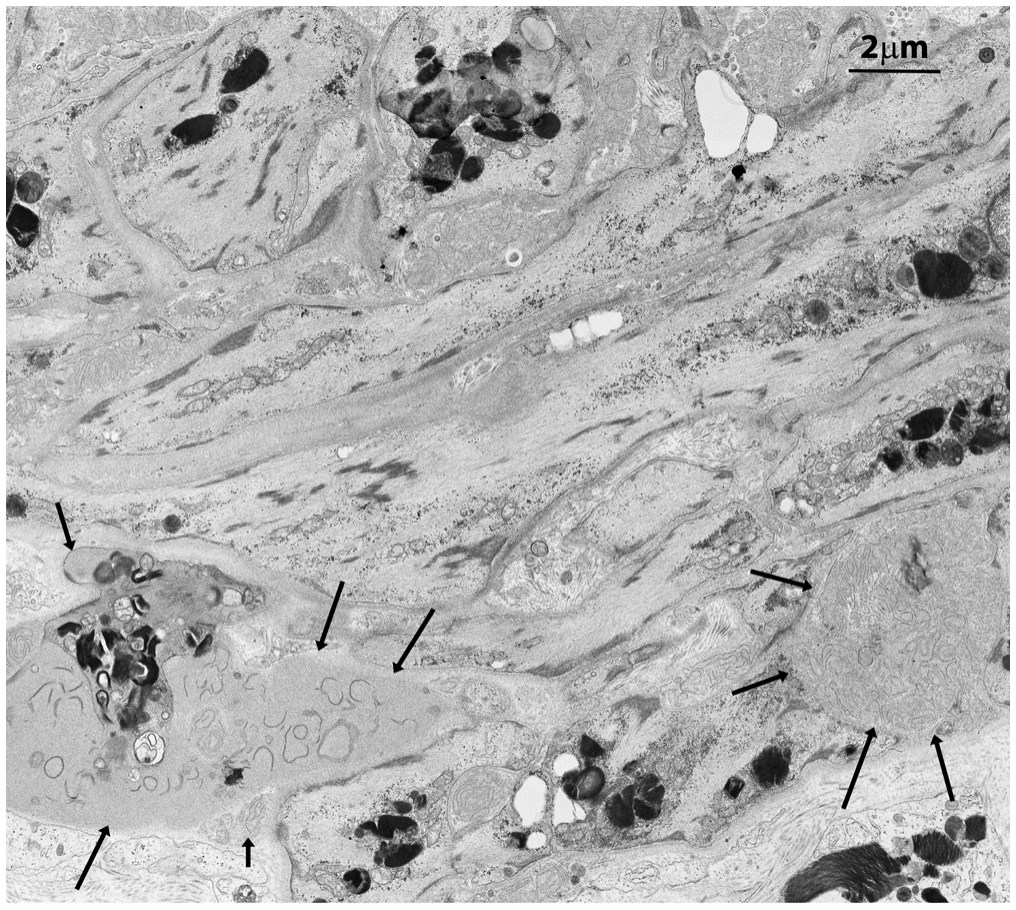
Squamous smooth muscle cells and fibroblasts at the basal pole of the epithelium of seminiferous tubules with two main features. They are organized in concentric layers and contain several GB bodies. In the extracellular space two exophers are also present (black arrows) with immature collagen fibers and extracellular GBs.

Testicular biopsies from FD (Fabry’s disease) patient were investigated for the accumulation of glycolipid bodies (GB) in various cells and subcellular compartments of testicular tissue. Observations using optical microscopy (OM) and transmission electron microscopy (TEM) revealed a substantial accumulation of GB3 as Glycolipid Bodies (GB) in the testicular vascular-connective stroma, contrasting with the tubular compartment, which appeared unaffected (**Figure 1**). Under OM (**Figure 1A**), GBs were identified as dark blue irregularly round cytoplasmic structures, while under TEM (**Figure 1B**), they appeared as zebra bodies with lamellar electron-dense inclusions or myeloid bodies. GB were predominantly found in stromal cells (**Figures 1**, **2**, **3**) but were absent in tubular seminiferous epithelium, Sertoli’s cells, and other seminiferous cell types (**Figures 1**, **2A**). Notably, GB were abundant in vessel wall cells, especially in smooth muscle cells of the media, fibroblasts, and pericytes in the adventitia, and were less prevalent in endothelial cells (**Figures 3**, **Supplementary Figure S3**). Furthermore, GB were also present in other interstitial cells, notably squamous smooth muscle cells, fibroblasts, and macrophages adjacent to the peritubular basement membrane (**Figures 1**, **2A**, **Supplementary Figure S3**).

In contrast to prior reports (11), Leydig’s cells appeared unaffected by GB accumulation, displaying well-preserved ultrastructural components, including the abundant vesicular smooth endoplasmic reticulum typical in cell producing sexual hormones (**Figure 2**). Occasionally, GB were found in the extracellular space, likely resulting from active secretion or release by damaged cells (**Figures 3**, **Supplementary Figure S3B**).

The thickening of tunica propria and of basement membrane in the tubules was observed, but Sertoli’s cells, spermatogonia, primary spermatocytes, and other cells of the seminiferous tubular epithelium did not show signs of GB accumulation. However, these cells exhibited severe damage, as indicated by reduced diameter, swollen or degenerated cytoplasm, and a paucity of primary spermatocytes and spermatozoa (**Figures 1**, **2**).

**Figure 4:**
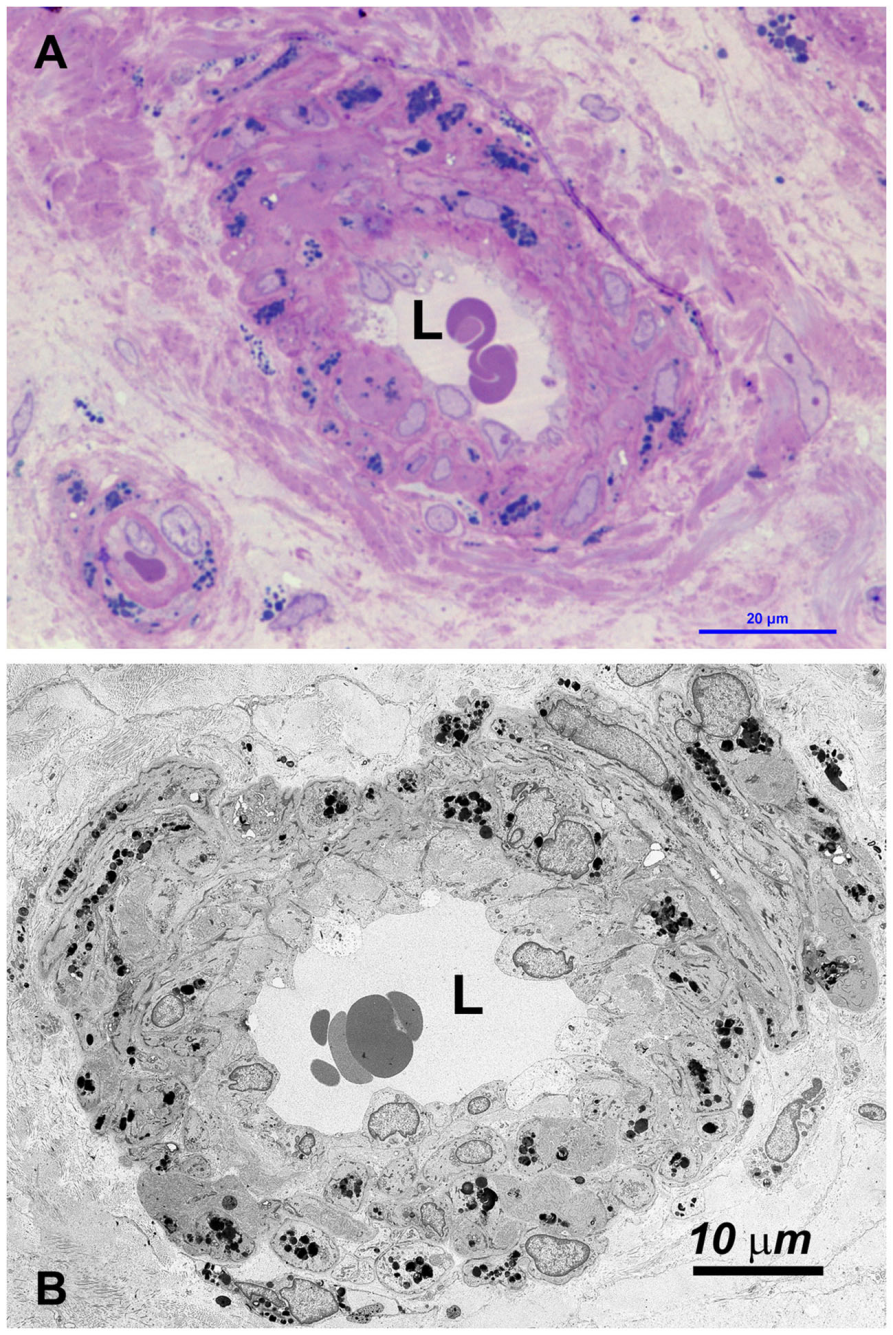
**(A)** OM: the wall of most small vessel (arterioles, venules and capillaries) is increased in thickness, due to swelling of cells and the accumulation of GBs (dark blue deposits). **(B)** TEM: GBs are prevalently localized in medial and adventitial layer, pericytes and occasionally in endothelial cells. The latter are swollen, with the cytoplasm protruding into the lumen (L) which may be reduced or occluded (see also **Supplementary Figure S4**).

Vessels were also severely affected in their different components, especially arterioles and capillaries (**Figures 4**, **Supplementary Figure S4**). Endothelial cells only rarely had GB but appeared swollen with nucleus protruding into the vessel lumen (**Figures 4**, **Supplementary Figure S4**). Endothelial basement membrane was thickened. Smooth muscle cells of media were the most affected by GB accumulation (**Figures 3**, **4**, **Supplementary Figure S4**). Adventitial layer of the vessel wall was substantially increased in thickness due to GB deposits and increase of mesenchymal cells (fibroblasts, fibrocytes, exopher secretion, increase of ECM and collagen (**Supplementary Figure S5**). For major clarity, the distribution and amount of GB in the different cellular types in the testis shown in TEM figures, are summarized in **Table 1**. These findings lead us to propose the hypothesis that GB3 accumulation in blood vessel walls, accompanied by reduced lumen, deposition of interstitial collagen and ECM, and thickening of the peritubular smooth muscle wall and basement membrane, may impede the diffusion of oxygen and nutrients, resulting in a hypoxic-inflammatory condition that change the expression/activation of HIF-1α and NFκB. The alteration of HIF-1α/NFκB axis, in turn, may damage the proper organization and function of the seminiferous cells leading to infertility.

**Table 1:** Glycolipid bodies (GB) distribution in testis tissue and cells.

	*Testis Cells*	*Glycolipid bodies*	*Figure*
1	Tubular germinative epithelium	Absent	1a, 1b
2	Sertoli’s cells	Absent	2a
3	Squamous smooth muscle cells	+++	3
4	Interstitial or stromal fibroblasts	++	1, 3, S3
5	Leydig’s cells	Absent	2a, 2b
6	Vessel pericytes	++	4b, S4
7	Vessel adventitial fibroblasts	++	4a, 4b
8	Vessel smooth muscle cells	++++	4a, 4b, S4, S5
9	Endothelial cells	++	4a, 4b, S4
10	Stromal extracellular space	++	S4, S5

### 3.2 Tubular and interstitial redistribution of HIF-1α, NFκB and VEGFA in testis of FD patient

**Figure 5:**
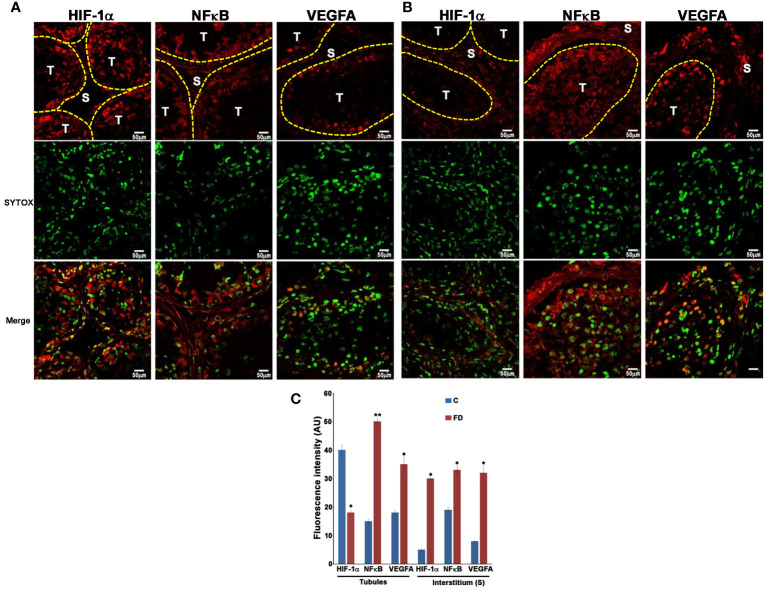
Expression of HIF-1α, NF**κ**B and VEGFA in control **(A)** and Fabry’s disease **(B)** patient testis as seen by Immunofluorescence. The image clearly shows the different distribution of HIF-1α, NF**κ**B and VEGFA in FD patient compared to the control. Nuclei were stained with SYTOX™ green. T, tubule; S, Stroma. **(C)** Quantification through Image J of the fluorescence intensity in **(A, B)** in the tubules and stroma in control and FD patient. AU, arbitrary units. *, statistically significant compared to the control. * p<0.05; ** p<0.01.

**Figure 6:**
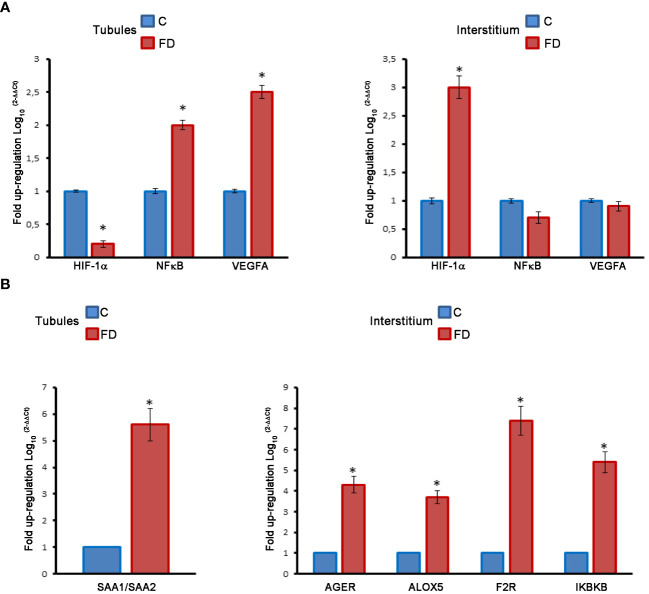
**(A)** Molecular analysis on LCMD fractions in control **(C)** and Fabry’s disease (FD) patient testis in tubules as compared to interstitial tissue. Expression and localization of HIF-1α, NF**κ**B and VEGFA. **(B)** Expression and localization of proinflammatory genes (SAA, AGER, ALOX5, F2R, IKBKB) in control and FD patient testis in tubules as compared to interstitial tissue. *, statistically significant compared to the control. * p<0.05.

In order to prove our hypothesis, we studied, by immunofluorescence, the expression and localization of HIF-1α, NFκB and VEGFA in sections of testicular biopsies from control and FD patient. Our results show an alteration of both localization and expression of these three proteins in FD compared to control. In fact, HIF-1α expression was localized in the tubules of control biopsy and in the interstitium in the FD patient (**Figures 5A, B**). Moreover, by staining the nuclei with SYTOX™ green, we observed that HIF-1α had a nuclear localization in the control and a cytoplasmic and perinuclear in the FD patient(s), probably indicating a different activation state (**Figures 5A, B**). On the other hand, NFκB showed an equal interstitial and tubular distribution in the control that strongly increased in FD (**Figure 5**). Finally, VEGFA expression increased in the tubules and interstitium of FD compared to the control (**Figure 5**). Immunofluorescence intensity in the tubules and interstitium of control and FD patients was quantified as reported in **Figure 5C**. To further prove the hypothesis and confirm immunofluorescence results, tubule and interstitium fractions were collected by LCMD tissue slices from control and FD patient. Such tubular and interstitial fractions underwent RT-PCR analysis to determine HIF-1α, NFκB and VEGFA gene expression. Results in **Figure 6A** show gene expression in FD compared to control. In the tubules we observed a decrease in HIF-1α and an increase in NFκB and VEGFA. In the interstitium, we observed an increase of HIF-1α in FD and a similar expression of NFκB and VEGFA between FD and controls (**Figure 6A**).

### 3.3 Activation of pro-inflammatory genes in the interstitial space of testicular biopsies

The same LCMD fractions obtained from testicular biopsies were processed to study the expression of a series of pro-inflammatory genes by RT-PCR. Interestingly most of the pro-inflammatory genes are expressed in the interstitium. In fact, in the tubules only the Serum Amyloid A1 and A2 (SAA1/SAA2) gene was overexpressed in FD compared to control (**Figure 6B**). Instead, in the interstitium, we observed an increase of Advanced glycosylated end product specific receptor (AGER), Arachidonate 5-lipoxygenase (ALOX5), Thrombin receptor or Coagulation Factor II Receptor (F2R), and Inhibitor of Nuclear Factor Kappa B Kinase Subunit Beta (IKBKB) (**Figure 6B**). The role of these genes in inflammatory-reparative response and the connection to the testis, where documented, is detailed in the Discussion.

## 4 Discussion

The presence of globotriaosylceramide (GB3) in the testis of individual with Fabry’s disease (FD) highlights an intriguing aspect of this condition (10). Notably, FD patient display normal sexual activity and hormonal levels (14), as it is also suggested by the unaffected Leydig’s cells (**Figure 2B**). However, reports indicate a spectrum of impacts on fertility among FD patients, ranging from average fertility to cases of azoospermia, alongside effects on semen morphology and function, leading to various degree of decrease in fertility (12–14).

Despite these observations, the morphological examination of GB3 accumulation in testicular biopsies has been limited, basically due to the rarity of this disease as well as the possibility to obtain tissue biopsies (10–12). In the attempt to overcome such limitations, murine and zebrafish models of Fabry’s disease have been generated. Murine models, however, do not fully recapitulate FD phenotype and mice, even if showing kidney and cardiac GB3 accumulations, have a normal lifetime (16, 17). On the other hand, zebrafish cannot accumulate GB3 lacking GB3 synthase and the GLA gene is on an autosomal chromosome (16, 17). However, this model has the advantage of the short growing rate and time to observe the effects of the disease in the kidney (16, 17). Interestingly, the zebrafish FD model has shown that kidney damages due to GLA absence arise even in the absence of GB3 accumulation, suggesting that other pathways parallel to those activated by GB3 might be present in the affected tissues (16, 17). In the case of humans, the abnormal accumulation of GB3 might obscure such other pathways (16, 17). Nevertheless, our restricted analysis suggests that the progressive accumulation of GB3 over time might underlie the varying quantitative morphological differences in the testicular tissue, contributing to diverse impacts on semen quality and fertility.

In our study, we conducted a comprehensive morphological, ultrastructural and molecular analysis of testicular biopsies from a 42-year-old FD patient. Surprisingly, GB3 accumulation was absent in the germinative epithelium, Sertoli’s and Leydig’s cells, but abundantly present in the interstitium, squamous smooth muscle cells, and blood vessel wall cells, albeit in varying amounts (**Figure 4** and **Table 1**). Furthermore, we observed a reduced diameter of the seminiferous tubular epithelium and Sertoli’s cells, significant thickening of the basement membrane and tunica propria, as well swollen or degenerated spermatogonia and spermatocytes (**Figure 1**).

We acknowledge the fact that the infertility we observed in the FD patient may be due to a genetic cause such as Y chromosome abnormalities or microdeletions (18). However, microdeletions are associated to high values of LH and FSH, whereas, as stated in the Methods, our FD patient has LH and FSH values in the normal range (19, 20). Moreover, in young age and before developing symptoms of Fabry's disease, the studied patient fathered two children suggesting, once again, that progressive cellular damage, more than microdeletions, is the cause for the sterility observed at an older age.

Sticking to our results, we propose a hypothesis of altered oxygen and nutrient diffusion from vessels, triggering a pro-inflammatory molecular response via the HIF-1α/NFκB axis (21–23). This pathogenetic pathway was substantiated through our immunofluorescence analysis, revealing an increased and altered distribution of HIF-1α, NFκB, and VEGFA. mRNA expression analyzed by RT-PCR on cell homogeneous tubular tissue fractions obtained by LCMD confirmed the decrease of HIF-1α and the increase of NFκB, and VEGFA. On the other hand, mRNA expression in the interstitium confirmed the increase of HIF-1α, but did not show the increase of NFκB, and VEGFA observed by immunofluorescence (**Figures 5**, **6**). This might be due to some post-transcriptional regulation of NFκB, and VEGFA mRNA or to an increased stability of NFκB, and VEGFA protein compared to their mRNA as also observed, in the case of VEGFA, by Arcondéguy (24). Overall, however, our results indicate a molecular activation of inflammatory-reparative responses in the testicular tissue of FD patient (**Figures 5**, **6**).

Hypoxia-inducible factor (HIF) is a critical transcription factor responding to decreased oxygen levels. Studies indicate that seminiferous tubules, similar to hypoxic niches, operate under reduced oxygen tension. Our findings suggest an altered expression of HIF-1α in the seminiferous tubules of FD patients, potentially disrupting oxygen homeostasis. Additionally, increased interstitial HIF-1α activation and nuclear accumulation were observed in FD patients (25–27). VEGFA, a downstream target of HIF-1α, associated with infertility in mice, exhibited increased expression in the seminiferous tubules of FD patients, consistent with our immunofluorescence and RT-PCR results (28) (**Figure 6A** **).**

Elevated HIF-1α and NFκB levels have been linked to inflammation, influencing the transcription of pro-inflammatory genes (29–31). Using LCMD, we precisely compared the level of some proinflammatory gene different expression in tubular and interstitial structures in biopsies from control and FD patients. Intriguingly, increased transcription of SAA1/SAA2 in the tubules and AGER, ALOX5, F2R, and IKBKB in the interstitium was observed (**Figure 6B**).

SAA1/SAA2, initially associated only with inflammation, is now recognized as a cytokine-like, immunologic, reparative and neoplastic protein, although its presence in the testis had not been reported previously (32). AGER is a receptor associated with cell damage and inflammation and has been observed in aged rats with implications for spermatogenesis (33, 34). ALOX5, widely expressed in various tissues including the testis, has recently been associated with inflammation and different forms of cell death, such as apoptosis, ferroptosis, and pyroptosis (panoptosis) (35). F2R is also associated with inflammation and triggers various pathobiological responses (36, 37). The function of IKBKB is to break IkB, the inhibitor of NFκB, thus favoring the nuclear accumulation of this proinflammatory transcription factor (38). Therefore, its increase in the interstitium of FD patient(s) stabilizes and increases nuclear accumulation of NFκB (**Figure 5** **).**

**Figure 7:**
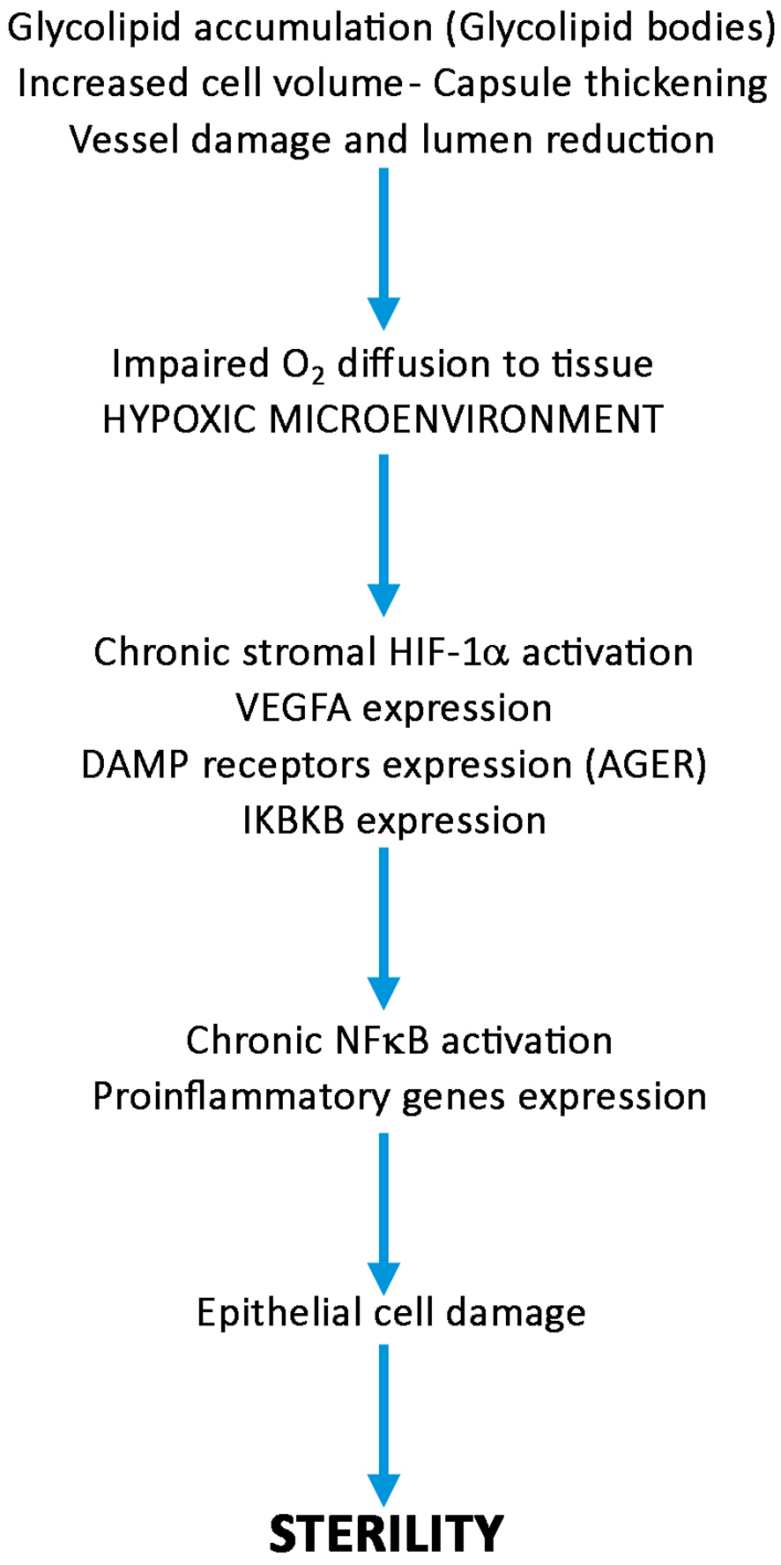
Diagram showing the possible role of HIF-1α/NF**κ**B axis activation in the pathogenesis of sterility in Fabry’s disease. Following progressive accumulation of glycolipid bodies, increased cell volume, vessel damage and thickening of peritubular capsule, oxygen diffusion to seminipherous epithelium is impaired, HIF-1α/NF**κ**B is activated producing cell damages leading to azoospermia or decrease in number and quality of spermatozoa.

Based on our findings, we draw a pathogenetic pathway activated by GB3 accumulation outlined on **Figure 7**. The accumulation of GB3 in the blood vessels and the thickening of the basal membrane likely restrict oxygen and nutrient supply, leading to the activation of HIF-1α, VEGFA and NFκB expression of pro-inflammatory genes, with cellular damage. These effects reduce cell vitality and, on the long run, fertility. In conclusion, our results suggest a link between GB accumulation, hypoxia, and an inflammatory-reparative response activated through the HIF/NFκB axis, contributing to infertility in FD patients and indicate new targets for possibly preventing sterility in FD patients by using HIF-1α/NFκB modulators and inflammasome inhibitors (39, 40).

## Data availability statement

The original contributions presented in the study are included in the article/**Supplementary Material**. Further inquiries can be directed to the corresponding author.

## Ethics statement

The studies involving humans were approved by Institutional Review Board University La Sapienza Opinion Number 6/2019. The studies were conducted in accordance with the local legislation and institutional requirements. The participants provided their written informed consent to participate in this study.

## Author contributions

LS: Investigation, Software, Writing – original draft. FB: Investigation, Software, Writing – original draft. MB: Investigation, Writing – review & editing. MA: Investigation, Writing – review & editing. AR: Investigation, Writing – review & editing. GP: Investigation, Writing – review & editing. MR: Software, Writing – original draft. MT: Software, Supervision, Writing – original draft, Writing – review & editing. AF: Software, Supervision, Writing – original draft, Writing – review & editing.
