# Corrigendum: Infertility in Fabry’s disease: role of hypoxia and inflammation in determining testicular damage

**DOI:** 10.3389/fendo.2024.1412673

**Published:** 2024-04-18

**Authors:** Luigi Sansone, Federica Barreca, Manuel Belli, Michele Aventaggiato, Andrea Russo, Giulietta A. Perrone, Matteo A. Russo, Marco Tafani, Andrea Frustaci

**Affiliations:** ^1^ Laboratory of Cellular and Molecular Pathology, IRCCS San Raffaele, Rome, Italy; ^2^ Department of Human Sciences and Promotion of the Quality of Life, San Raffaele Roma Open University, Rome, Italy; ^3^ Laboratory of Cellular and Molecular Pathology, Department of Experimental Medicine, Sapienza University, Rome, Italy; ^4^ UOC of Pathologic Anatomy, IRCCS Regina Elena National Cancer Institute, Rome, Italy; ^5^ UOC of Pathologic Anatomy, San Filippo Neri Hospital, Rome, Italy

**Keywords:** Fabry’s disease, hypoxia, inflammation, cellular and molecular rehabilitation, testicular damage, new therapeutic targets for infertility rehabilitation

In the published article, there was an error in affiliation 4. Instead of “Department of Pathologic Anatomy, IRCCS Regina Elena, Rome, Italy”, it should be “UOC of Pathologic Anatomy, IRCCS Regina Elena National Cancer Institute, Rome, Italy”.

The authors apologize for this error and state that this does not change the scientific conclusions of the article in any way. The original article has been updated.

