# Supplementary figures and images for: Infertility in Fabry’s Disease: role of hypoxia and inflammation in determining testicular damage
Source: Front Endocrinol (Lausanne). 2024 Feb 22;15:1340188. doi: 10.3389/fendo.2024.1340188 (PMC10917934; doi:10.3389/fendo.2024.1340188)

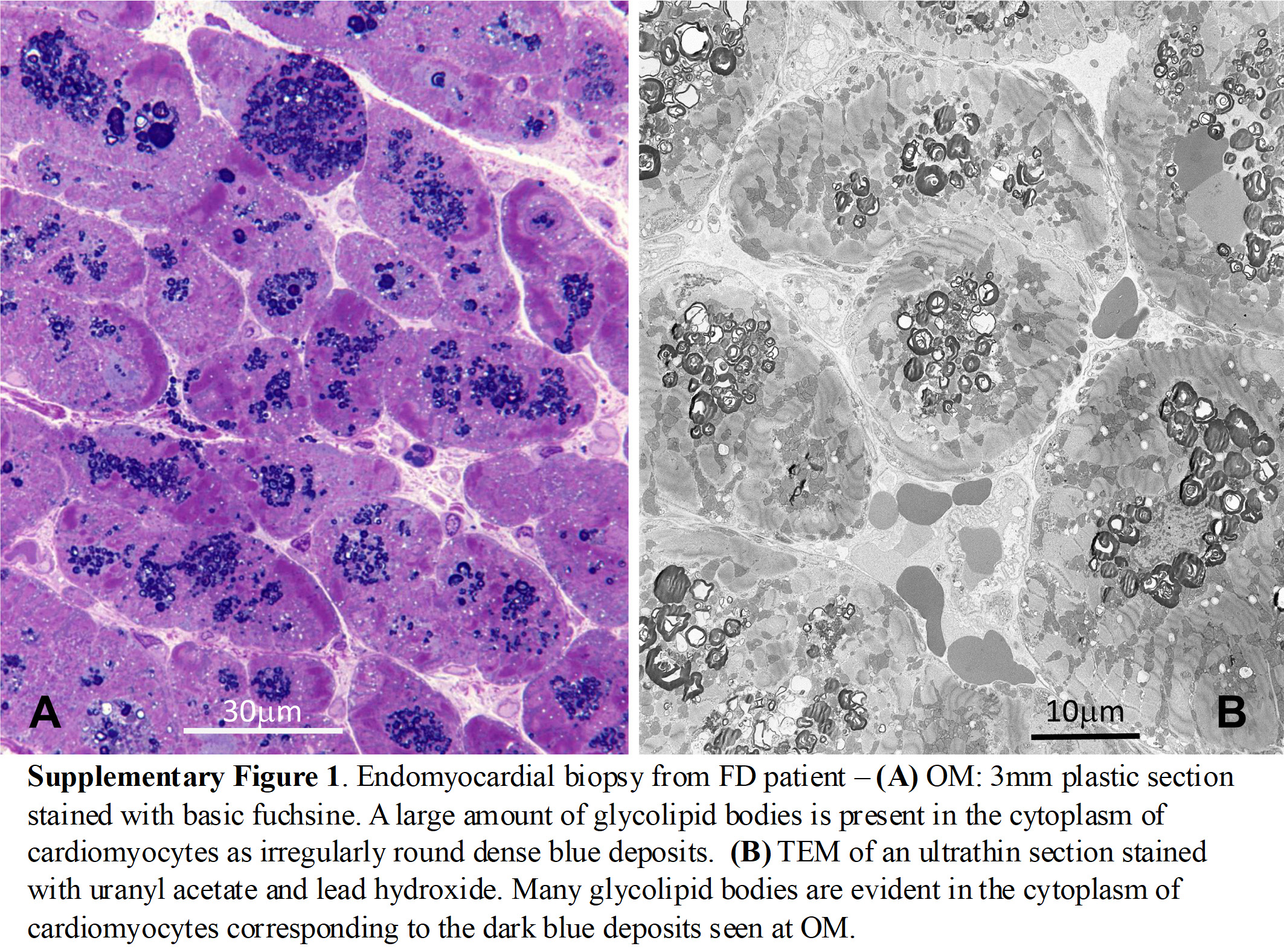

Supplement: Supplementary file 1 [file Image_1.jpeg]

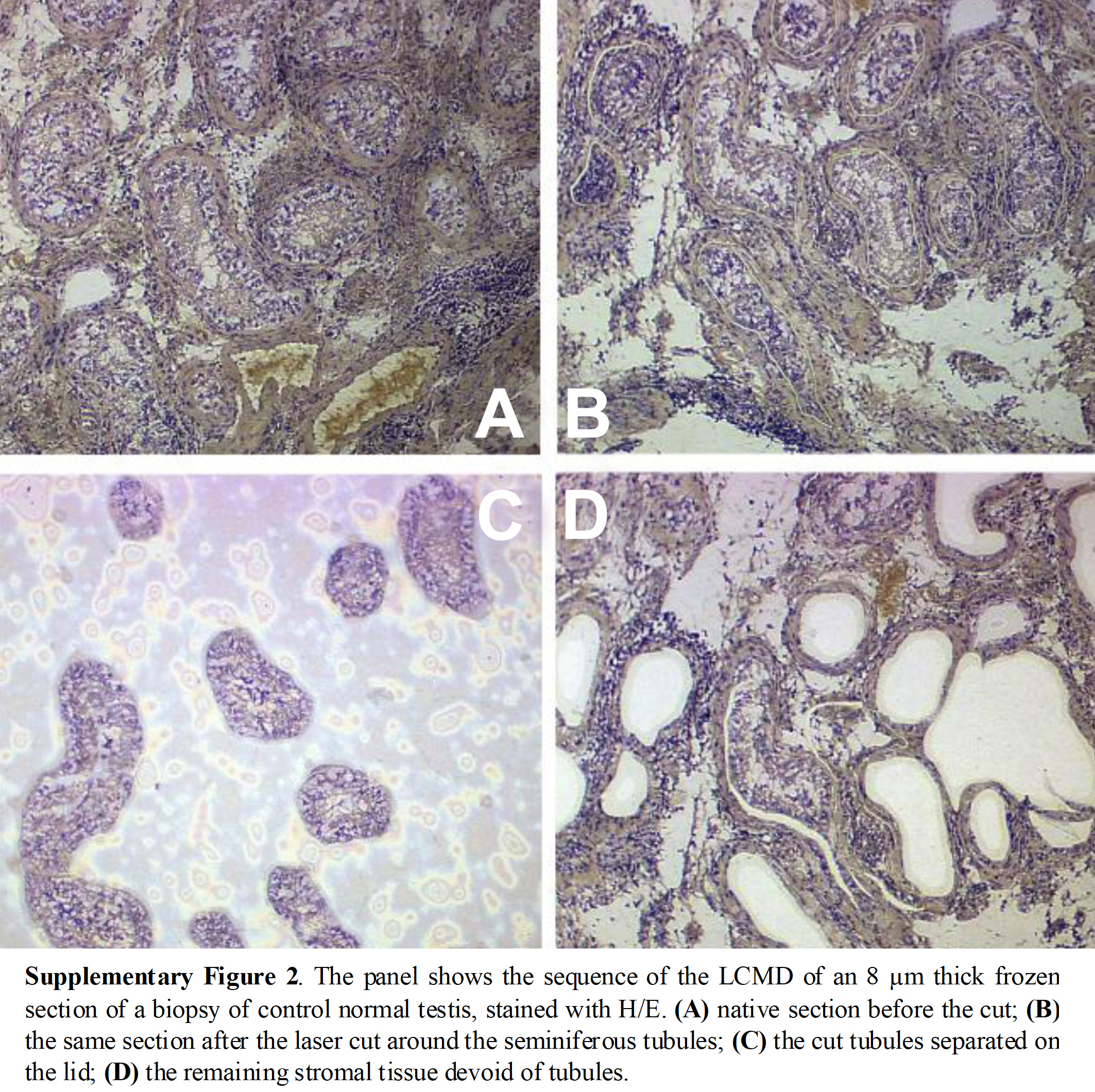

Supplement: Supplementary file 2 [file Image_2.jpeg]

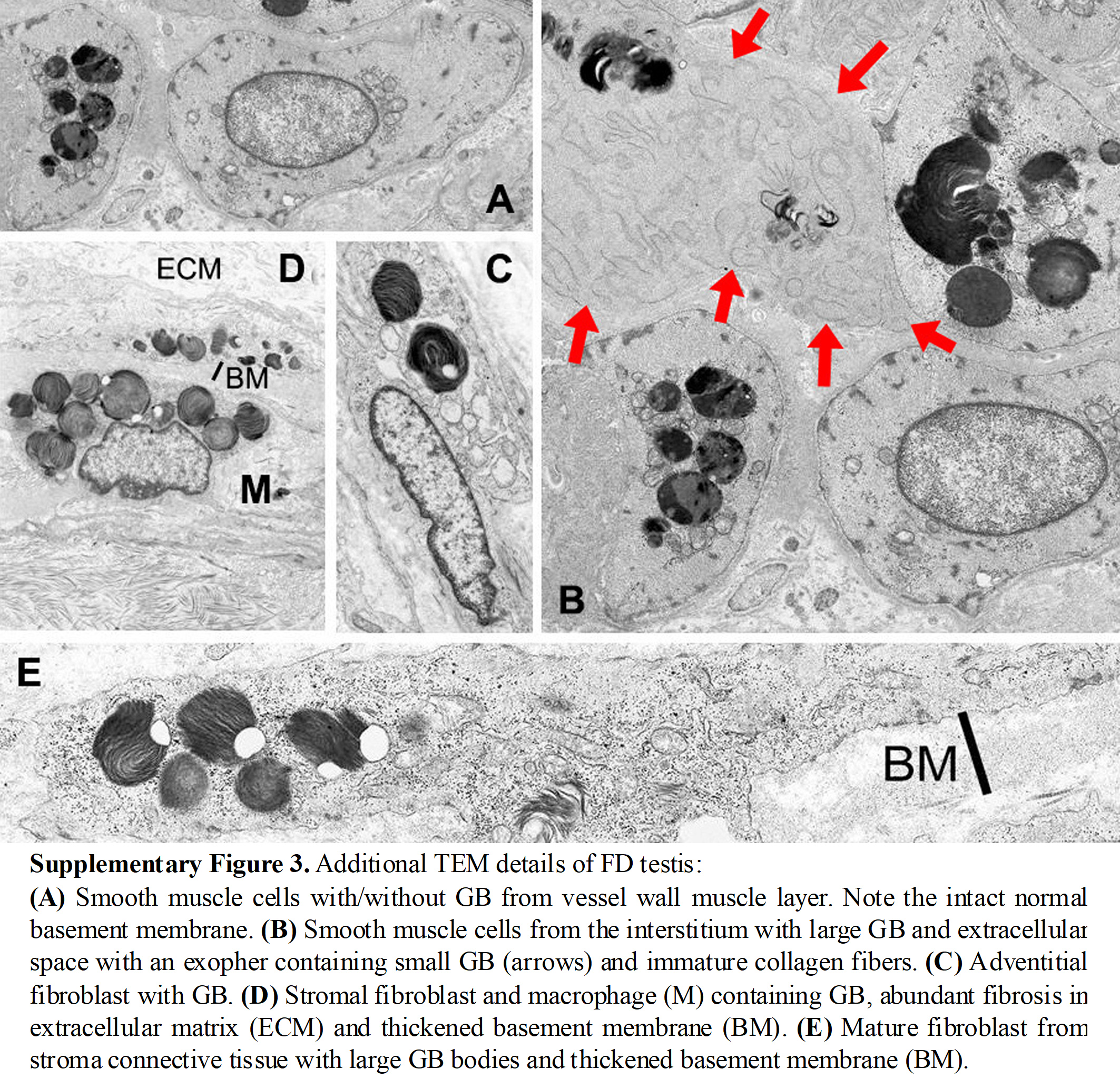

Supplement: Supplementary file 3 [file Image_3.jpeg]

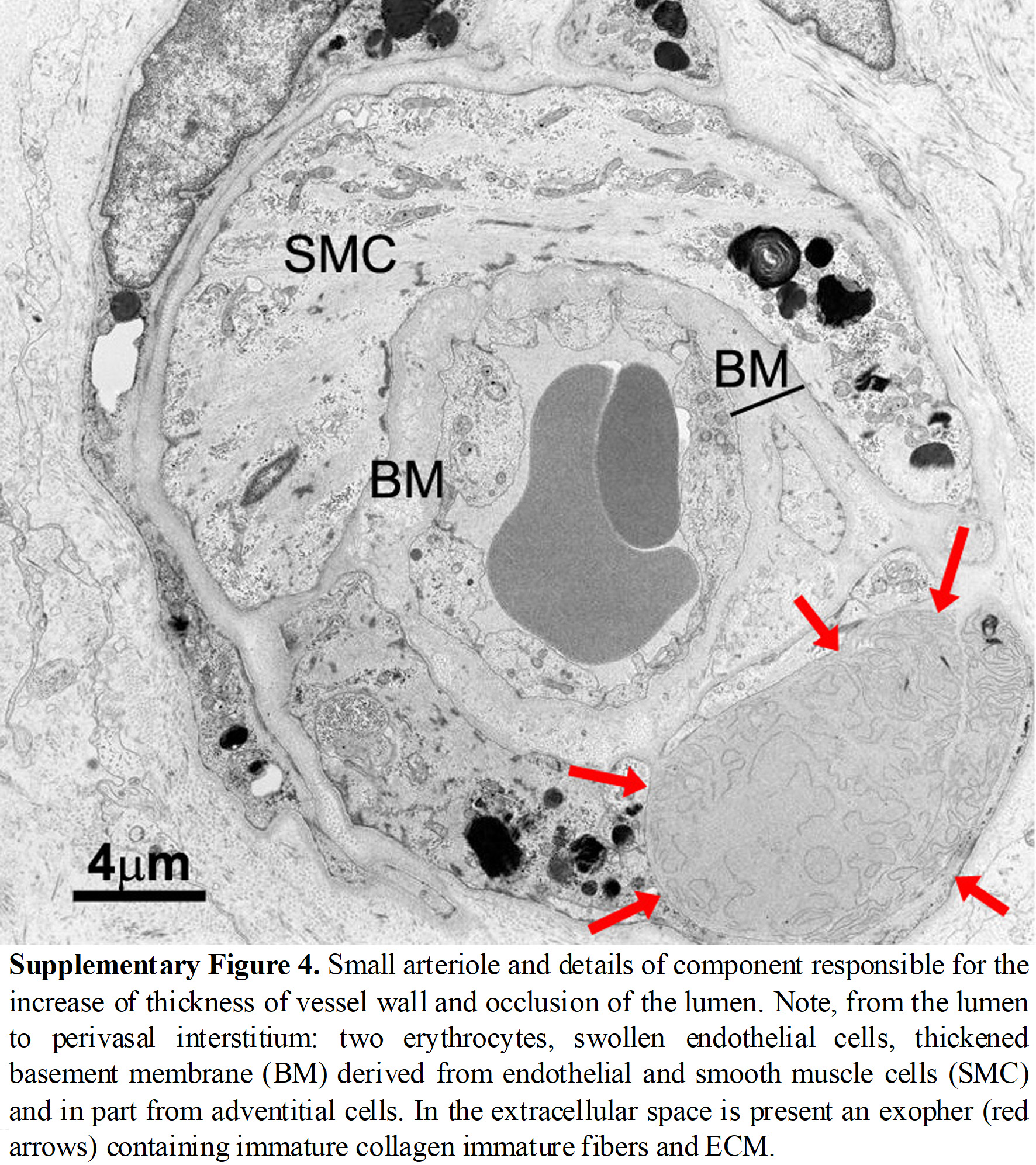

Supplement: Supplementary file 4 [file Image_4.jpeg]

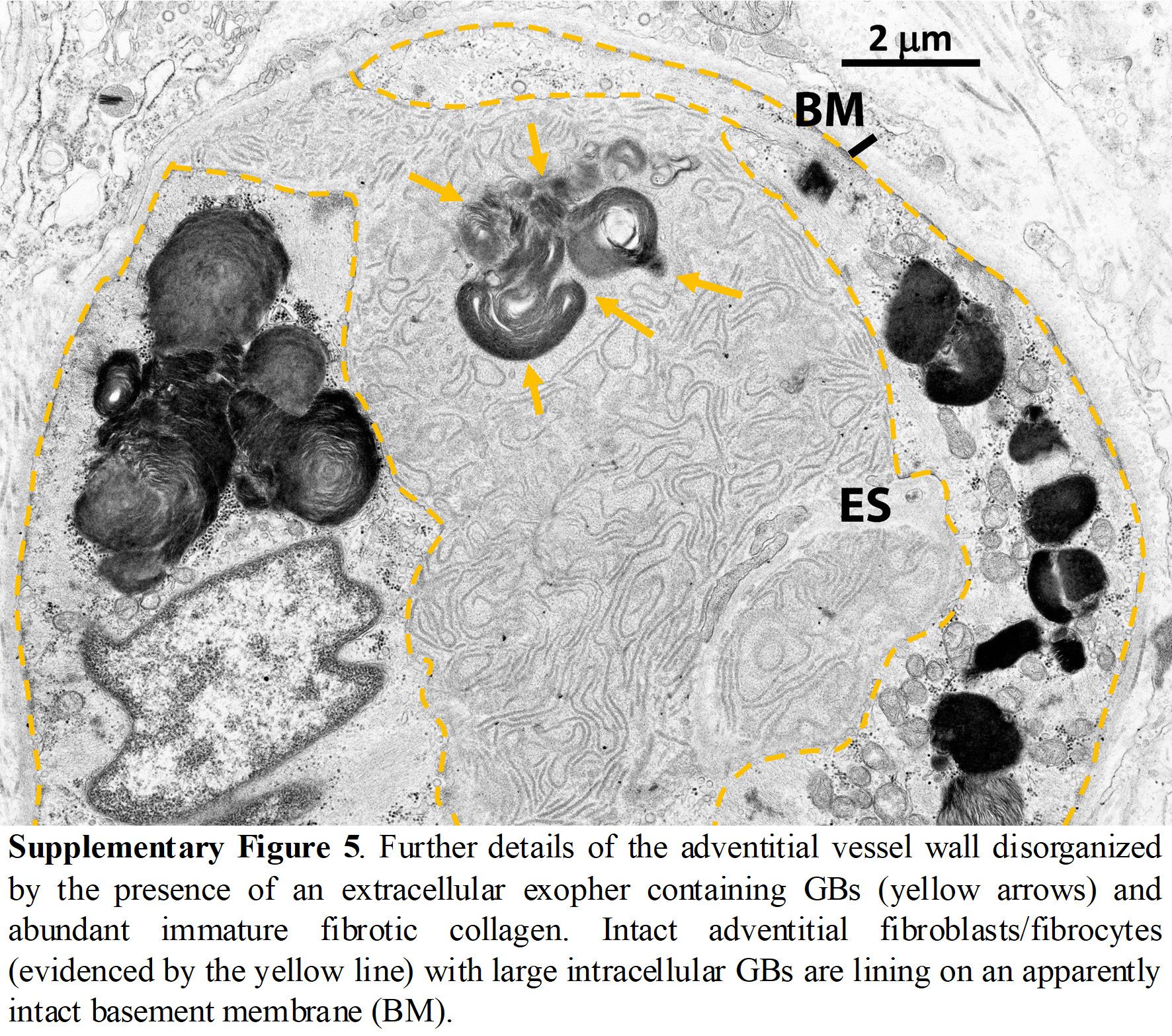

Supplement: Supplementary file 5 [file Image_5.jpeg]
